# A rare case of familial restrictive cardiomyopathy, with mutations in MYH7 and ABCC9 genes

**DOI:** 10.15190/d.2019.12

**Published:** 2019-09-30

**Authors:** Oana Neagoe, Anda Ciobanu, Rodica Diaconu, Oana Mirea, Ionuț Donoiu, Constantin Militaru

**Affiliations:** Department of Cardiology, Emergency County Hospital, Craiova, Romania; Department of Cardiology, University of Medicine and Pharmacy of Craiova, Romania

**Keywords:** Familial restrictive cardiomyopathy, genetic testing, cardiac transplantation.

## Abstract

Restrictive cardiomyopathy is the least common type of cardiomyopathy, being defined by diastolic dysfunction and often unimpaired systolic function. Restrictive cardiomyopathies can be classified as familial or non-familial. Patients with familial restrictive cardiomyopathy can develop signs and symptoms of this condition anytime from childhood to adulthood. The evolution of the disease is towards signs and symptoms of pulmonary and systemic congestion and, without treatment, there is a five-year mortality rate of approximately 30% in these patients. We discuss the case of a 43-year-old patient diagnosed with familial restrictive cardiomyopathy with positive genetic tests for mutations of MYH7 gene and ABCC9 gene, who was first hospitalized in 2011 for palpitations. The echocardiography performed in evolution showed a continuous alteration of right ventricle function, without important differences of left ventricular function.  She developed heart failure symptoms six years after diagnosis and she had seven hospitalizations in the past two years, currently with an increasing need of diuretics and persistent hepatic dysfunction. Cardiac transplantation or left ventricular assist device therapy should be considered in patients with severe heart failure symptoms and no longer effective treatment. However, elevated pulmonary vascular resistance excludes the patient from cardiac transplantation.

## **INTRODUCTION**

Restrictive cardiomyopathies are defined as a group of myocardial diseases that is hallmarked by abnormal diastolic function due to myocardial fibrosis, infiltration or endomyocardial scarring^1,2^. The main hemodynamic features are excessive rigidity of ventricular walls, high ventricular filling pressures, either low or normal ventricular volume along with often unimpaired systolic function and normal or increased left ventricular wall thickness.

According to cause, restrictive cardio-myopathies can be classified as non-infiltrative (e.g. Idiopathic cardiomyopathy), infiltrative (e.g. Amyloidosis, Sarcoidosis), storage disease (e.g. Hemochromatosis) and endomyocardial (e.g. Endomyocardial fibrosis; as a result of radiation, toxic effects of anthracycline)^3,4^. Moreover, according to ESC, restrictive cardiomyopathies can be classified as familial (with autosomal dominant pattern in most of the cases or autosomal recessive pattern in some of them) and non-familial^5^.

Patients with familial restrictive cardiomyopathy can develop signs and symptoms of this condition anytime from childhood to adulthood. When patients first become symptomatic as adults, they usually develop dyspnea, fatigue, exercise intolerance, palpitations or dizziness. Atrial fibrillation is very often showed by ECG. The further evolution of the disease would be towards signs and symptoms of pulmonary and systemic congestion and, without treatment, there is a five-year mortality rate of approximately 30% in these patients^6,7^.

Diagnosis of familial restrictive cardio-myopathy implies not only medical history, physical examination, blood tests, chest X-ray, ECG, multimodality imaging (echocardiography, cardiac CT, MRI, nuclear imaging), but also genetic testing^5-8^. The main differential diagnosis that needs to be taken into consideration is constrictive pericarditis.

Most common genetic defects identified in familial restrictive cardiomyopathy are TNNI3 (one of the major causes of this condition), TNNT2, MYH7, ACTC1, TPM1, MYL3, MYL2. The MYH7 gene is encoding beta-myosin heavy chain, a protein found in heart muscle and in type I skeletal muscle fibers (found, for example, in muscles involved in posture, such as neck muscles)^9-12^. The beta-myosin heavy chain is involved in forming a larger protein called type II myosin, which is a part of sarcomeres (the basic units of muscle contraction)^9-12^. There have been studies that suggested an important contribution for mutations in MYH7 in dilated cardiomyopathy, hypertrophic cardiomyopathy (up to 35% of all cases), peripartum cardiomyopathy, non-compaction cardiomyopathy and congenital heart defects. However, it has not been possible yet to establish the exact mechanism of action or the impact over the onset, evolution or prognosis of the cardiomyopathies in patients who develop mutations of this gene^13-17^.

Here, we describe the case of a 43-year-old patient diagnosed with familial restrictive cardiomyopathy, with mutations in MYH7 and ABCC9 genes.

## **CASE REPORT**

The patient filled in an informed consent for participating to the study.

The study presents the case of a 43-year-old female patient, who was first hospitalized in 2011 in the Cardiology Clinic of Emergency County Hospital of Craiova for palpitations. We noted a positive familial history for restrictive cardiomyopathy in her mother’s sister, deceased at the age of 60 years old, who was diagnosed only by echocardiography. However, there were no genetic tests performed in her case. Moreover, our patient related a history of cardiovascular disease in her mother and maternal grandfather. However, they are no longer alive, and we have no medical documents concerning their pathology. Furthermore, we decided to perform echocardiography to patient’s 20-years-old asymptomatic son and there were no pathological changes found. Even though we recommended genetic testing in his case, it has not been performed yet.

**Figure 1:**
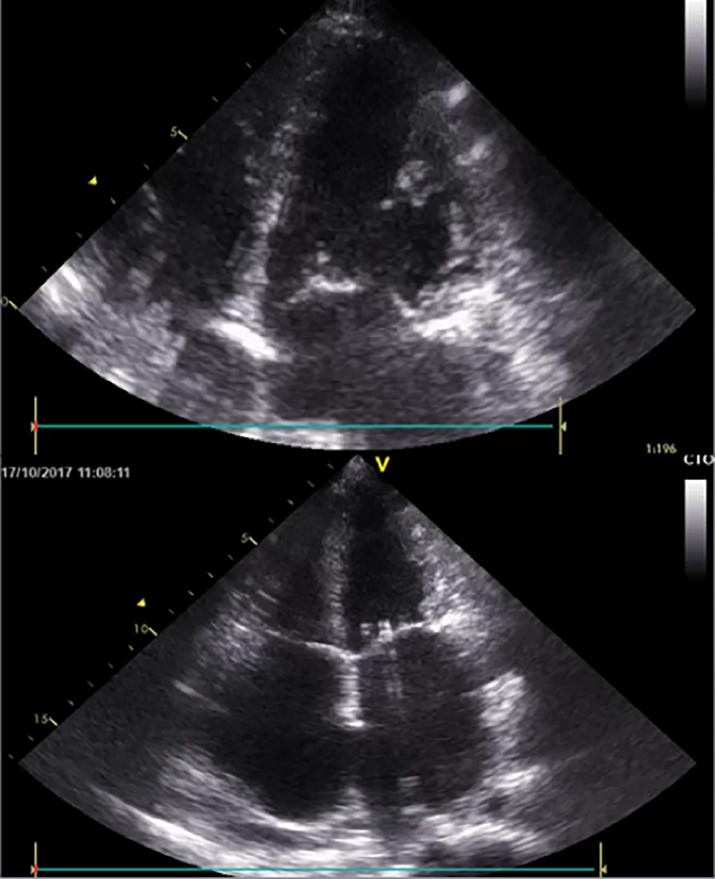
Echocardiography: Apical Four Chamber view, typical aspect of restrictive cardiomyopathy, severe biatrial enlargement with normal ventricular volume

She could not mention any other medical history. The ECG revealed atrial fibrillation, with QRS axis at 90 degrees, Q wave in DIII, negative T wave in DI, DII, DIII, aVF, V1-V6. Echocardiography revealed normal dimensions of aortic root and ascending aorta, normal diameter of left ventricle, left ventricular volume slightly reduced, normal LVEF (55%), mild mitral regurgitation, severe left atrial enlargement, normal right ventricular diameter and slight right ventricular longitudinal systolic dysfunction, severe right atrial enlargement, mild tricuspid regurgitation, moderate pulmonary hypertension (**Figure 1**). We started oral anticoagulation and we decided to attempt electric conversion to sinus rhythm, which was successful.

At that point, the diagnosis of restrictive cardiomyopathy has already been put in discussion and the next investigation that we recommended was a cardiac MRI, which showed reduced left ventricular volume, normal systolic function (LVEF=62%), normal left ventricular wall thickness, without any pathological changes of regional contractility. Certain trabeculations were described in the left ventricle, without meeting the criteria for non-compaction cardiomyopathy. Moreover, we did not identify abnormal iron overload neither in the myocardium, nor in the liver. In addition, there was normal right ventricular volume with normal systolic function (RVEF=55%), without changes in regional contractility or wall aneurysms. Both left and right atrium had increased volume. Mitral and tricuspid mild regurgitations were present. After contrast administration, there were no signs of microvascular obstruction in early time. Moreover, there was no evidence of late enhancement. Furthermore, a coronary angiography was performed showing that all coronary arteries were permeable.

Further tests included: immunofixation tests, storage diseases testing, complete profile of biologic markers for autoimmune diseases, abdominal adipose tissue biopsy puncture (stained with Congo Red dye) and genetic tests.

All of them were negative, except the genetic testing which identified a heterozygous mutation in MYH7 c.2162G>A (p.R721K) gene located on the 14q11.2 chromosome, with a probably pathogenic significance, not being reported before in the literature according to ACMG guidelines and being located in a mutational hotspot without benign variants. The phenotype described for MYH7 gene is associated with Hypertrophic Cardiomyopathy 1 (OMIM 160760), a disease with autosomal dominant transmission. Moreover, the genetic testing was positive for a heterozygous mutation in ABCC9 c.3557G>A (p.R1186Q) gene, located on the 12p12.1 chromosome, a variant of uncertain significance, likewise not being reported before in the literature. The phenotype described for ABCC gene mutations is associated with Familial atrial fibrillation-12 (OMIM 614050), a disease with autosomal dominant transmission.

In 2014, she returns for another episode of atrial fibrillation. This time, the transesophageal echocardiography revealed left atrial thrombosis. Thus, the conversion was postponed and unsuccessful, when it finally was attempted. The 24-hour Holter ECG monitoring diagnosed permanent atrial fibrillation, with a minimum HR of 53 beats per minute and a maximum HR of 113 beats per minute, with rare ventricular extrasystoles and without RR intervals longer than 2 seconds. The treatment that was prescribed at home was with bisoprolol 5 mg, acenocoumarol 2 mg, digoxin 0,125 mg, furosemide 40 mg, spironolactone 25 mg, omeprazole 20 mg.

In 2017, our patient was admitted to hospital for dyspnea at low physical exertion, edema of lower extremities and severe fatigue. Clinical examination revealed pallor, perioral cyanosis, scleral jaundice, BP of 90/60 mmHg and HR of 120 beats per minute, arrhythmic heart beats, abolished vesicular breathing sounds at the base of the right lung, spontaneous oxygen saturation level of 90%, pain in the right hypochondrium spontaneously and on mild palpation, enlarged liver and distended jugular veins.

Blood tests revealed hyponatremia (Na^+^=119 mmol/L), hypokalemia (K^+^=3,4 mmol/L), hypoproteinemia (serum total proteins=4,9 g/dL, serum albumin=2,6 g/dL), high levels of bilirubin (direct bilirubin=0,53 mg/dL, indirect bilirubin=1,5 mg/dL, total bilirubin=2,08 mg/dL), INR=3,7 and NT-proBNP=9484 mg/dL, with all other blood tests within normal limits. Echocardiography revealed in addition a mild/moderate mitral regurgitation, mild/moderate tricuspid regurgitation, dilated inferior vena cava without inspiratory collapse, intracavitary spontaneous contrast and at the level of inferior vena cava. The abdominal ultrasound showed liver with diffuse inhomogeneous, hyperechoic echostructure, with slightly irregular contour and dilated hepatic veins, moderate quantity of right pleural fluid (confirmed as well by the chest X-ray) and ascites. During the hospitalization she received intravenous diuretics, spironolactone, bisoprolol, acenocumarol, human albumin, hydro-electrolyte rebalancing solutions and right thoracentesis was performed with extraction of 700 mL of fluid, with favorable evolution. The treatment that was recommended at discharge was: acenocoumarol 1 mg/day, bisoprolol 10 mg/day, furosemide 80 mg/day, spironolactone 50 mg/day, omeprazole 20 mg/day.

The patient was referred to a specialized center for heart transplantation. However, she decided to delay the consultation.

In the past 3 years she was hospitalized several times for heart failure symptoms and persistent liver dysfunction, with the need of increasing the daily dose of diuretics to 160 mg furosemide and reintroducing digoxin 0,125 mg/day.

## **DISCUSSION**

Restrictive cardiomyopathy is the least common type of cardiomyopathy (after dilated cardiomyopathy and hypertrophic cardiomyopathy), representing approximately 5% of pediatric cardiomyopathies^18^. It is more frequent in males. In almost 50% of the patients it is secondary to a systemic disorder, the most common being amyloidosis^19^. Concerning the prevalence of familial restrictive cardiomyopathy, it remains unknown for the moment.

The treatment of restrictive cardiomyopathy patients is not specific. It is mainly symptom-driven and involves diuretics, aldosterone antagonists, vasodilators and ACEIs. There are patients with familial amyloidosis who have responded to novel therapies, such as molecular and drug therapies that target abnormal protein production. However, in most patients diagnosed with familial restrictive cardiomyopathy, the severity of heart failure symptoms and the lack of response to treatment could be indications for cardiac transplantation^20,21^. Furthermore, there are certain patients for whom permanent pacing or left ventricular assist device therapy should be considered.

**Figure 2:**
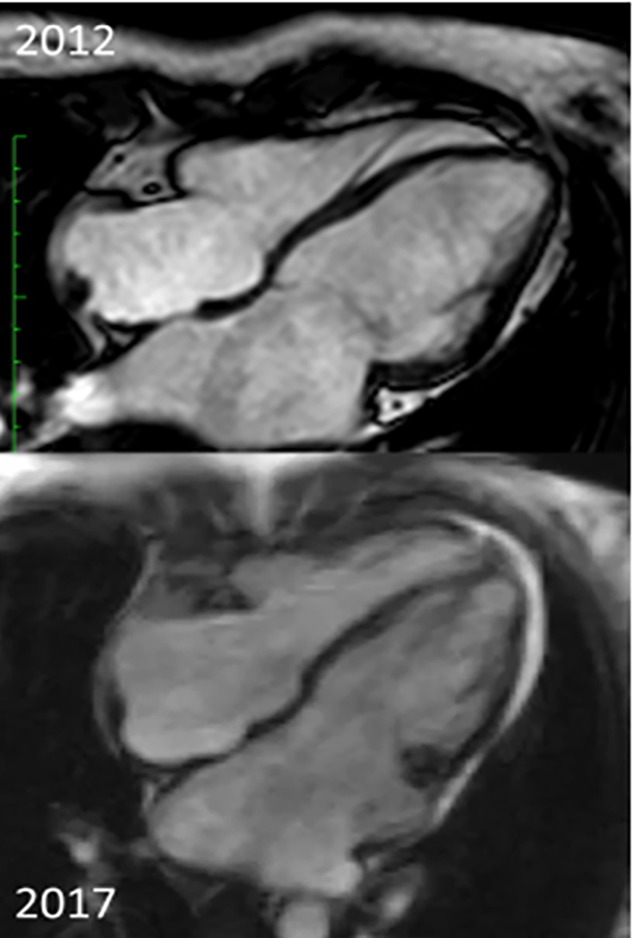
A comparison between cardiac MRI performed in 2012 and 2017

Our patient developed heart failure symptoms six years after diagnosis and the symptomatic treatment seems to have no longer the same effectiveness. In our clinic, she had 8 hospitalizations and the last 7 were recorded in the past two years, currently with an increasing need of diuretics and persistent liver dysfunction. The cardiac MRI performed in 2017 showed no additional changes than the one performed in 2012 (**Figure 2**). However, the echocardiography showed mainly a continuous alteration of right ventricle function, without important differences in the left ventricular systolic function (**Table 1**).

**Table 1:** A comparison between values of echocardiographic parameters recorded in 2017 and 2019

Echocardiographic parameter	2017	2019
LVEF (%)	60	55
Indexed EDV LV (ml/m2)	48	36
Indexed EDV LA (mL/m2)	75	80
E wave (m/s)	0,78	0,7
Deceleration time (ms)	133	131
LV septal S' (m/s)	0,04	0,04
RV wall S' (m/s)	0,07	0,07
TAPSE (mm)	20	13
Estimated sPAP (mmHg)	32	43

As we mentioned above, our patient decided to delay a decision concerning cardiac transplantation. For the moment, in her case, it could still be considered. However, elevated pulmonary vascular resistance excludes patient from cardiac transplantation. Thus, she needs to be closely followed−up and, if needed, it should be taken into consideration ventricular mechanical support therapy as a bridge to transplantation^22^.

## **CONCLUSION**

Restrictive cardiomyopathy is a rare type of cardiomyopathy that can have many causes. Multimodality imaging, along with complete blood tests and genetic testing are the key in establishing the correct diagnosis. Management is still a challenge; the main approach is symptomatic treatment. Cardiac transplantation or left ventricular assist device therapy should be considered in patients with severe heart failure symptoms and no longer effective treatment.

## KEY POINTS

**◊** **Familial restrictive cardiomyopathy is a rare disease that could be linked with mutations in MYH7 gene**

**◊** **Diagnosis is established through a multimodality approach including cardiac imaging and genetic testing**

**◊** **There is no specific treatment for familial restrictive cardiomyopathy**
